# Metataxonomic and Immunological Analysis of Feces from Children with or without Phelan–McDermid Syndrome

**DOI:** 10.3390/microorganisms12102006

**Published:** 2024-10-02

**Authors:** Claudio Alba, Carmen Herranz, Miguel A. Monroy, Alberto Aragón, Rubén Jurado, David Díaz-Regañón, César Sánchez, Mar Tolín, Carmen Miranda, Bárbara Gómez-Taylor, Francisca Sempere, Guillermo Álvarez-Calatayud, Juan M. Rodríguez

**Affiliations:** 1 Department Nutrition and Food Science, Complutense University of Madrid, 28040 Madrid, Spain; drdiazreganon@ucm.es (D.D.-R.); jmrodrig@ucm.es (J.M.R.); 2 Instituto Pluridisciplinar, Complutense University of Madrid, 28040 Madrid, Spain; alberara@ucm.es (A.A.); rubenjur@ucm.es (R.J.); 3 Centro de Salud Lucero, 28047 Madrid, Spain; monroytapiador@gmail.com; 4 Department Galenic Pharmacy and Food Technology, Complutense University of Madrid, 28040 Madrid, Spain; 5 Departamento de Nutrición Humana, Universidad Católica de Valencia, 46001 Valencia, Spain; sangoros@hotmail.com (C.S.); marth81@gmail.com (M.T.); mcmirandina@gmail.com (C.M.); bgomeztaylor@gmail.com (B.G.-T.); francisca.sempere@ucv.es (F.S.); 6 Department Pediatric Gastroenterology, Hospital Universitario Gregorio Marañón, 28007 Madrid, Spain; galvarezcalatayud@gmail.com

**Keywords:** Phelan–McDermid syndrome, autism spectrum disorder, microbiota, feces, immunoprofiling, short-chain fatty acids

## Abstract

Phelan–McDermid syndrome (PMS) is a neurodevelopmental disorder characterized by a developmental delay and autism spectrum disorder (ASD)-like behaviors. Emerging research suggests a link between gut microbiota and neuropsychiatric conditions, including PMS. This study aimed to investigate the fecal microbiota and immune profiles of children with PMS compared to healthy controls. Fecal samples were collected from children diagnosed with PMS and age-matched healthy controls. The bacterial composition was analyzed using 16S rRNA gene sequencing, while short-chain fatty acids (SCFAs) were quantified through gas chromatography. Immunological profiling was conducted using a multiplex cytokine assay. Significant differences were observed in the gut microbiota composition between PMS patients and controls, including a lower abundance of key bacterial genera such as *Faecalibacterium* and *Agathobacter* in PMS patients. SCFA levels were also reduced in PMS patients. Immunological analysis revealed higher levels of several pro-inflammatory cytokines in the PMS group, although these differences were not statistically significant. The findings indicate that children with PMS have distinct gut microbiota and SCFA profiles, which may contribute to the gastrointestinal and neurodevelopmental symptoms observed in this syndrome. These results suggest potential avenues for microbiota-targeted therapies in PMS.

## 1. Introduction

Rare diseases include many neurodevelopmental disorders that are usually diagnosed during infancy [1]. Among them, Phelan–McDermid syndrome (PMS) is a de novo genetic disorder frequently caused by deletions in the terminal end of chromosome 22 (22q13.3) or mutations affecting the SHANK3 gene [2,3]. The SHANK3 protein is crucial for neural communication and development because of its role as a scaffolding protein within the postsynaptic densities of excitatory glutamatergic synapses [4,5]. As a result, inactivation of the SHANK3 gene in mouse models leads to reduced postsynaptic densities and disrupted synaptic transmission [6,7]. More recently, it has been found that chromosomal rearrangements that do not affect SHANK3 may result in the same general phenotype (SHANK3-unrelated PMS) [8]. This fact may be explained because of the functional overlapping between SHANK3 and other genes that contribute to PMS in gene functions associated with neurodevelopment, synaptic formation, and inflammation [9].

The prevalence of PMS seems to be very low, ranging from 2.5 to 10 cases per million births [10]. A recent survey of large cohorts showed that PMS may have the lowest prevalence rates among de novo genetic neurodevelopment disorders, together with Fragile X and Angelman syndromes [11]. However, PMS may be largely underdiagnosed because of the absence of a genetic diagnosis, the existence of SHANK3-unrelated PMS and the difficulties in obtaining a proper sequence of the GC-rich SHANK3 gene [12]. 

PMS may account for about 1–2% of autism spectrum disorder (ASD) cases [13,14,15] while most PMS patients also have a diagnosis of ASD. There are some similarities and, also, some differences between PMS and idiopathic ASD (iASD) [10], including a higher tendency of PMS patients to have more severe medical complications and intellectual disability [16]. Although the symptoms and their severity may vary among PMS patients, the most common ones include global neurodevelopmental delays, hypotonia and absent or delayed speech.

In addition, gastrointestinal (GI)-related symptoms are common comorbidities of both ASD and PMS patients, including constipation, diarrhea, fecal incontinence, abdominal pain, gastroesophageal reflux, vomiting and/or rumination disorder [12,17,18,19,20]. GI dysfunction is a major concern for families and caregivers of PMS patients [21,22] and may arise, at least partly, because of the involvement of SHANK3 and other PMS-associated genes in the neurological control of digestive functions, in the functionality of enterocytes and in reinforcing the intestinal barrier [23,24,25].

It has been described that the risk of suffering from GI symptoms among children with ASD seems to be linked to alterations in the gut microbiota [26,27,28,29], and correlate with the severity of ASD [30,31,32]. Recent research has increasingly focused on the role of the gut microbiota in neurodevelopmental and neuropsychiatric disorders [33,34,35]. The gut microbiota comprises a complex community of microorganisms residing in the gastrointestinal tract, which significantly influences various bodily functions, including immune modulation, metabolic processes, and brain development and behavior through the gut–brain axis [36]. This bidirectional communication network involves multiple pathways, including the nervous system, the immune system, and metabolic signaling [37,38].

Alterations in the composition of the gut microbiota (dysbiosis) have the potential to negatively affect gut motility and permeability, the production of metabolites that are relevant for gut health, such as short chain fatty acids, and immune function, promoting local and systemic inflammation, which may contribute to the appearance or worsening of both the gastrointestinal and neurological symptoms observed in neurodevelopmental disorders [36,39,40]. In other words, alterations in the gut microbiota can potentially contribute to the pathophysiology of conditions like ASD [41,42] or PMS. Overall, these observations have spurred interest in exploring how these microbial communities differ in children with neurodevelopmental disorders compared to healthy controls.

Although the scientific literature regarding potential relationships between PMS and the gut microbiota is very scarce, it has been suggested that the GI symptoms frequently exhibited by children with PMS might be linked to their altered gut microbiota [13]. This potential relationship underscores the need to investigate the gut microbiota composition in PMS patients and how it correlates with their symptoms. Investigating the gut microbiota in PMS can provide insights into the extent and nature of this dysbiosis, and whether it mirrors the patterns observed in ASD. This is crucial for developing targeted interventions, which have shown promise in modulating the gut microbiota and alleviating symptoms in ASD [33,43,44,45,46,47,48]. In this context, the principal aim of this study was to assess the fecal bacterial microbiota and immune profiles of children with PMS and to compare them with those of healthy age-paired ones.

## 2. Materials and Methods

### 2.1. Participants

Participants included children with a diagnosis of PMS, confirmed by the Spanish PMS Association (https://22q13.org.es/, accessed on 5 September 2024), and healthy volunteer controls selected based on the absence of disease symptoms and lack of chronic medication use. Lack of use of antibiotics, probiotics, or prebiotics in the four weeks prior to sample collection was mandatory in both groups. For all the participants, an informed consent form was signed by their parents or guardians. This study was approved by the Ethics Committee of the Hospital Gregorio Marañón (Madrid, Spain) (protocol: TPB-PhM/2022; date of approval: 10 March 2023).

### 2.2. Sample Collection and DNA Extraction

Fecal samples were collected from all participants and stored at −20 °C until analysis. DNA was extracted from the samples using QIAamp DNA Stool Mini Kit (QIAgen, Hilden, Germany) according to the manufacturer’s instructions. The concentration and purity of the extracted DNA were measured using a NanoDrop spectrophotometer.

### 2.3. Amplification, Sequencing of the 16S rRNA Gene and Bioinformatic Analysis

The hypervariable V3-V4 region of the bacterial 16S rRNA gene was amplified using PCR. Equimolar concentrations of the universal primers S-D-Bact-0341-b-S-17 and S-D-Bact-0785-a-A-21 were used. PCR products were pooled in equimolar DNA concentrations and run on an agarose gel. Bands were extracted and purified using QIAEX II Gel Extraction Kit (QIAgen) and quantified with PicoGreen (BMG Labtech, Jena, Germany). Aliquots of the amplicons were sequenced using the paired-end sequencing protocol on the Illumina MiSeq sequencer (Illumina Inc., San Diego, CA, USA) at the Scientific Park of Madrid, Spain. Sequences were demultiplexed using Illumina software (version 2.6.2.3) following the manufacturer’s guidelines.

Bioinformatic analyses were conducted using a combination of QIIME 2 2021.1 and R software (version 3.5.1, https://www.r-project.org/, accessed on 4 February 2020). The DADA2 pipeline was used for sequence cleaning and filtering: forward reads were truncated at position 285 with the first 12 nucleotides trimmed, and reverse reads were truncated at position 240 with the first 9 nucleotides trimmed to discard positions with a mean nucleotide quality below Q20. Taxonomy data were assigned to amplicon sequence variants (ASVs) using the q2-feature-classifier classify-sklearn naïve Bayes classifier against the SILVA 138.1 reference database. The Shannon and Simpson diversity indices were calculated using the R vegan package to estimate alpha diversity, considering both the number and evenness of microbial species, with the Wilcoxon rank-sum test used to identify statistical differences between groups. Beta diversity was studied using principal coordinates analysis (PCoA) to visualize patterns through a distance matrix. Quantitative and qualitative analyses were performed using Bray–Curtis and binary Jaccard indices, respectively. Permutational multivariate analysis of variance (PERMANOVA) with 999 permutations was employed.

### 2.4. Short-Chain Fatty Acid (SCFA) Analysis

The quantification of three short-chain fatty acids (acetic, propionic and butyric acids) in the fecal samples was performed by gas chromatography as previously described [49]. Briefly, 100 μL of a 1:10 dilution of fecal matter (*w*/*v*) in phosphate-buffered saline (PBS; pH 7.4) was supplemented with 100 μL of 2-ethyl butyric acid (Sigma-Aldrich, St. Louis, MO, USA) as an internal standard (1 mg/mL in methanol) and acidified with 100 μL of 20% formic acid (*v*/*v*). The acidic mixture was then extracted with 1 mL of methanol and centrifuged for 10 min at 15,800× *g*. The supernatants were stored at −20 °C until analysis using a GC apparatus. The system comprised a 6890 GC injection module (Agilent Technologies, Santa Clara, CA, USA) with an HP-FFAP (30 m × 0.250 mm × 0.25 μm) column (Agilent Technologies), operating in split mode with a split ratio of 1:20. The injection volume was 1 μL, with injector and detector temperatures set at 240 °C and 250 °C, respectively. The column oven temperature was initially set at 110 °C, then increased at a rate of 6 °C/min to 170 °C, and subsequently at 25 °C/min to 240 °C, resulting in a total GC run time of 18 min. Helium was used as the carrier gas at a constant flow rate of 1.3 mL/min. The chromatographic system featured a flame ionization detector (FID), and data acquisition and processing were carried out using ChemStation Agilent software v. LTS 01.11 (Agilent Technologies).

### 2.5. Immunological Analysis

To perform the immunological analysis, the fecal samples were processed as described previously [50]. The concentrations of a wide array of human immune factors (FGF basic, eotaxin, G-CSF, GM-CSF, IFNγ, IL-1β, IL-1ra, IL-2, IL-4, IL-5, IL-6, IL-7, IL-8, IL-9, IL10, IL12 (p70), IL-13, IL-15, IL-17A, IP-10, MCP-1, MIP-1α, MIP-1β, PDGF-BB, RANTES, TNF-α, VEGF) were determined using the Bio-Plex Pro Human Cytokine 27-plex Assay kit (Bio-Rad, Hercules, CA, USA) in the Bio-Plex 200 instrument (Bio-Rad). Every assay was run in duplicate and standard curves were performed for each analyte.

### 2.6. Statistical Analysis

Statistical analyses were conducted using R software. Descriptive statistics summarized the demographic and clinical characteristics of the study population. For normally distributed data, the mean values were reported, and the 2-tailed Student’s *t*-test was used for comparisons, while for non-normally distributed variables, the median values were reported and the Wilcoxon Mann–Whitney U test was employed for PMS patients and controls. Differences were considered statistically significant at *p* < 0.05. Alpha diversity was assessed using both the Shannon and Simpson diversity indices, which accounted for the abundance and evenness of microbial species. Beta diversity was illustrated using principal coordinates analysis (PCoA) derived from distance matrices. The Bray–Curtis index was employed for quantitative analysis, while the binary Jaccard index was used for qualitative analysis. Statistical differences in beta diversity were evaluated using PERMANOVA with 999 permutations, identifying significant differences at *p* < 0.05.

## 3. Results

### 3.1. Participant Demographics and Clinical Characteristics

A total of 42 children with Phelan–McDermid syndrome (PMS) and 22 healthy controls were included in the study. The demographic and clinical characteristics of the participants are summarized in Table 1. The PMS group consisted of 54.8% males, with a median age of 11 years (range: 7–15 years), whereas the healthy control group comprised 50% males, with a median age of 10 years (range: 6–14 years). Other relevant characteristics, such as BMI and presence of gastrointestinal (GI) symptoms, are also detailed in Table 1.

### 3.2. Metataxonomic Analysis

**Figure 1:**
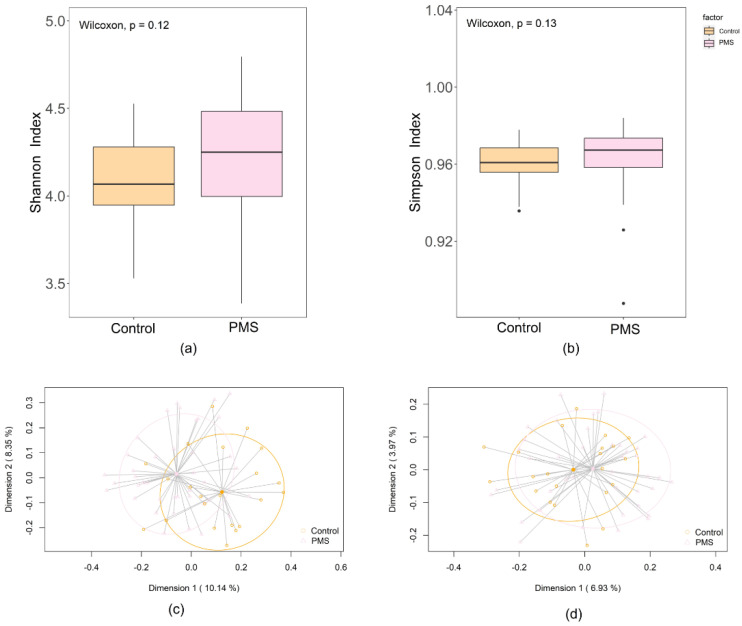
Metataxonomic profiles of fecal samples of healthy controls (Control; orange), and patients with Phelan–McDermid syndrome (PMS; pink). (**a**) Comparison of alpha diversity at the ASV level calculated using the Shannon index between both groups of patients. (**b**) Comparison of alpha diversity at the ASV level calculated using the Simpson index between the groups. Principal coordinate analysis (PCoA) plots of bacterial profiles at the genus level based on (**c**) the Bray–Curtis dissimilarity index in each group and (**d**) Jaccard’s coefficient for binary data (presence or absence). The values on each axis label in graphs (**c**,**d**) represent the percentage of the total variance explained by that axis.

A total of 62 out of the 64 samples collected in this study were sequenced. Two samples from PMS children were excluded from sequencing because of the low DNA yield and the lack of amplification after the first PCR round, respectively. Overall, the remaining 62 samples yielded a total of 13,671,053 reads. The sequencing depth ranged from a minimum of 144,265 to a maximum of 415,179 reads per sample. The median number of reads for the control group was 221,063, with an interquartile range (IQR) of 205,319 to 244,717.75, while for the PMS cases, the median number of reads was 211,182.5, with an IQR of 196,299 to 228,883. The gut bacterial diversity assessed using the Shannon diversity index did not reveal significant differences between PMS patients (median [IQR] = 4.25 [4.00–4.48]) and healthy controls (median [IQR] = 4.07 [3.95–4.28]) (*p* = 0.12). Similarly, the Simpson index in PMS patients (0.97 [0.96–0.97]) and in controls (0.96 [0.96–0.97]) was very similar (*p* = 0.13). In contrast to alpha diversity, the study revealed significant differences in beta diversity between both groups, being significantly lower in the PMS group. Using Bray–Curtis distance metrics, the differences in microbial community composition were highly significant (*p* < 0.001). Similarly, the Jaccard index, which measures the presence or absence of species, indicated significant differences between the two groups (*p* = 0.003) (Figure 1).

Differences in the relative abundance of some bacterial genera were also detected when comparing PMS children and healthy controls (Table 2). PMS patients had a lower median relative abundance of *Faecalibacterium* (PMS patients: 2.13% [IQR: 1.16–7.35], control group: 9.82% [IQR: 4.93–12.36]; *p* = 0.00037), *Bacteroides* (PMS patients: 1.51% [IQR: 0.36–4.66], control group: 4.59% [IQR: 2.57–7.33], *p* = 0.004), *Subdoligranulum* (PMS patients: 5.57% [IQR: 2.97–9.7], control group: 8.24% [IQR: 6.42–15.05]; *p* = 0.024), *Agathobacter* (PMS patients: 1.33% [IQR: 0.43–4.72], control group: 9.58% [IQR: 1.8–12.61]; *p* = 0.011), *Alistipes* (PMS patients: 0.55% [IQR: 0.16–2.24], control group: 1.41% [IQR: 0.45–3.79]; *p* = 0.038) and *Roseburia* (PMS patients: 0.83% [IQR: 0.37–1.59], control group: 1.59% [IQR: 0.95–3.24]; *p* = 0.0066). In contrast, the median relative abundance of *Bifidobacterium* was higher in PMS patients (1.98% [IQR: 0.98–6.38] than in controls (1.22% [IQR: 0.85–2.53]), although in this case the differences did not reach statistical significance (*p* = 0.3).

### 3.3. Short-Chain Fatty Acid (SCFA) Analysis

The concentrations of SCFAs (acetate, propionate and butyrate) were higher in the feces of the control group than in those of the PMS group (Table 3). The differences between the PMS and the control samples were statistically significant for all SCFAs (*p* < 0.001).

### 3.4. Immunological Analysis

PDGF-bb, IL-12 (p70) and RANTES were the immune factors with the highest detection frequency (>90% in the samples from both groups), followed by IL-1ra and IL-17 (>70%), and Il-6, IL-10 and IFN-γ (>50%). On the contrary, VEGF, MIP-1b, MCP-1, FGF basic, eotaxin, IL-8 and IL-4 showed a very low frequency of detection in this study (<10%) (Table 4).

In relation to the concentration of those immune factors detected in at least 30% of the samples of both groups, the means in the PMS group were higher than in the control group for IL-1β (2.7 ng/L versus 1.0 ng/L), IL-1ra (994 ng/L versus 832 ng/L), IL-10 (4.1 ng/L versus 2.9 ng/L), IL-17 (6.0 ng/L versus 3.6 ng/L), and TNF-α (29 ng/L versus 14 ng/L). However, the interindividual variability in the values for these immune compounds was high, and consequently, such differences did not reach statistical significance (*p* > 0.05). The mean values for the rest of the immune factors detected in at least 30% of the samples of both groups were very similar in the two study groups.

## 4. Discussion

In recent years, several studies have addressed the microbiomes among individuals diagnosed with ASD [51,52,53,54]. However, very few of them have been focused on patients with PMS, a condition that includes ASD-like behaviors and that is mainly caused by deletions in 22q13, the genetic region containing SHANK3 and other genes [17,55,56]. In this study, the fecal metataxonomic, SCFA and immunological profiles of a cohort of PMS patients were assessed and compared with those of a control population.

In relation to the fecal metataxonomic analysis, the main significant differences in PMS samples compared to controls were, overall, a lower β-diversity and a lower relative abundance of the phylum Bacteroidota and the genera *Faecalibacterium*, *Agathobacter*, *Alistipes* and *Roseburia*, and a higher relative abundance of the phylum Actinomycetota and the genus *Bifidobacterium*. Previously, two studies dealing with *Shank3* knock-out (KO) mice displaying ASD-associated behaviors revealed an altered gastrointestinal morphology, which was accompanied by a dysbiosis state [25,57]. Both studies described that the abundance of Actinomycetota was significantly higher in feces from *Shank3αβ* KO mice than in those from controls. More specifically, Sauer et al. [25] found that the genus *Bifidobacterium* was one of the drivers of such increase, while Morton et al. [58] described the existence of ASD-associated amino acid, carbohydrate and lipid profiles predominantly encoded by microbial species in the genera *Bifidobacterium*. Such results agree with the higher abundance of the phylum Actinomycetota and the genus *Bifidobacterium* observed in our study. Interestingly, a significant increase in the phylum Actinomycetota has also been reported in the feces of human patients with ASD [52].

In addition, we found a decrease in the abundance of the genus *Bacteroides* in the feces of PMS patients. This genus is also characterized by its high ability to produce SCFAs (especially propionate), to downregulate the systemic levels of pro-inflammatory cytokines, such as IL-6, TNF-α or IL-1β [59], and to protect against systemic inflammation induced by lipopolysaccharide (LPS) [60,61]. However, metataxonomic studies comparing ASD cases and controls have provided conflicting results regarding the phylum Bacteroidota and the genus *Bacteroides*. Some of them have reported an increase in the relative abundance of this phylum in ASD patients [51,52,62,63]; in the same direction, it has been described that the treatment of newborn mice with *Bacteroides fragilis* led to increased repetitive behaviors and social dysfunction in males but not in females [64]. In contrast, but in agreement with our results, other works found the contrary [54], including the observation that the administration of *B. fragilis* to pregnant C57BL/6N mice attenuated abnormal communicative and repetitive behaviors among the offspring [33], and that there was a decreased abundance of this genus in samples of duodenal mucosa collected from ASD subjects [65].

Such inconsistencies among the bacterial taxa alterations found in different studies targeting subjects with ASD are widespread, affecting different genera [66]. In relation to *Faecalibacterium*, the decreased abundance in PMS samples agrees with the data provided on ASD subjects by three previous studies [51,53,67] but is in disagreement with those reported by other researchers [27,51,52,54,68,69]. Another study found that a reduced abundance of *Faecalibacterium* and *Agathobacter* (formerly, Eubacterium) was associated with sleep disorders in children with ASD [70]. Decreases in the abundance of *Agathobacter*, *Alistipes*, *Roseburia* and *Subdoligranulum* have also been reported in other works [71,72,73,74]. Thus, while almost all these studies have identified alterations in the composition of the microbiome in ASD patients, they have failed in finding a minimal consensus regarding the specific bacterial genera that are commonly affected. The situation is even worse when dealing with PMS individuals because of the low number of studies specifically focused on this population.

Further functional studies (e.g., metabolomic) involving larger and well-defined populations are necessary to reach a minimum consensus regarding specific microbiota-associated functional shifts in iASD and PMS hosts, and to elucidate if there may be microbial signatures that differentiate these two types of neurodevelopmental disorders. In this context, a decrease in the levels of SCFAs or in the abundance of genes linked to the production of SCFAs has been consistently found in ASD metagenomes [70,72,75], a relevant finding since these bacterial products enhance the integrity of both the gut epithelium and the blood–brain barrier [76]. In the study by Liu et al. [75], both the ASD and the control group had similar dietary sources of butyrate, and therefore, the authors concluded that the primary drivers of SCFA changes were the differences in microbial taxa.

In this work, we detected significant differences in the levels of three SCFAs (butyrate, acetate and propionate) when PMS samples were compared to those provided by healthy individuals. This is a relevant finding since these bacterial products are relevant for several key biological processes. At the intestinal level, they enhance the integrity of the gut epithelium, controlling the entry of inflammatory compounds to the bloodstream [76]. Intestinal SCFAs obtain systemic access [77], crossing the blood–brain barrier while enhancing its integrity [76,78]. Furthermore, SCFAs are recognized as key mediators in the interaction between the gut microbiota and the immune system, helping to regulate the balance between anti-inflammatory and pro-inflammatory responses and to maintain immune homeostasis [79]. This immunomodulatory function takes place not only in the gut, but also in other host locations, including the central nervous system [80]. In fact, SCFAs are crucial in the regulation of the gut–brain axis, influencing central nervous system functions such as cell-to-cell communication, neurotransmitter production and release, microglia activation, mitochondrial activity, gene expression and early neural system development [81,82]. More specifically, butyrate has been shown to consolidate long-term memory [83], and to stimulate neurogenesis, neural proliferation and expression of brain-derived neurotrophic factor in murine models [84,85,86]. Propionate contributes to the healthy development of the brain and healthy behavior [33,87], and its levels are altered in ASD cases [77]; finally, supplementing with acetate improves social deficits and modifies transcriptional regulation in the prefrontal cortex of mice lacking SHANK3 [88].

Previous studies have shown an increase in inflammatory markers, particularly in IL-6, in ASD patients and in ASD animal models [89,90,91,92,93,94]. IL-6 immunofluorescence was not higher in the neural tissue of *Shank3αβ* KO mice compared to controls but was significantly increased in blood vessels [62]. In our study, the fecal levels of some ASD-associated cytokines, such as IL-1β, IFN-γ and TNF-α, were higher in the PMS samples than in the controls, but no statistically significant difference between both groups was detected, most probably because of the high interindividual variability. High levels of IL-1β and TNF-α may induce detrimental immune responses in the brain through binding to the brain endothelial cells [95]. In contrast, the mean values for IL-6 were similar in the two groups. Our results may also have been influenced by the type of biological sample (feces) or the host species, and therefore might not be comparable with those obtained from blood or in mice. Regardless, all of these facts indicate that reduced levels of SCFA-producing bacteria and/or SCFAs in the gut ecosystem may contribute to the development or worsening of the gastrointestinal and neurodevelopmental symptoms that characterize PMS patients because of their profound impact on the gut microbiota–neuroimmune crosstalk [96].

This study has several limitations that should be considered when interpreting the results, including the relatively small sample size, its cross-sectional design and the lack of a functional analysis of the gut microbiota. The small sample size is reflective of the rarity of PMS; nevertheless, the sample includes a high proportion of the Spanish PMS population, where the first study carried out to evaluate the prevalence of this disorder provided a rate (4 × 10^−4^/10,000 inhabitants) [97] that was much lower than those previously reported in other countries (2.5 to 10 cases per million births) [10]. Thus, this fact limits the generalizability of our findings and may reduce the statistical power to detect subtle differences between groups. Additionally, the cross-sectional design precludes us from drawing conclusions about causality or the temporal relationship between microbiota alterations and symptom progression in PMS. Finally, the lack of a functional analysis of the gut microbiota prevents us from discussing its actual role in the health or disease outcomes of PMS. However, this study has allowed the establishment of a solid Spanish PMS cohort, opening up the possibility of increasing our very limited knowledge about this disorder. In fact, ongoing work aims, first, to further elucidate the differential roles that the microbiota may play in PMS patients compared to controls, through omics approaches and data mining, and, secondly, to develop microbiota-based strategies that could improve the health and well-being of children with PMS.

## Figures and Tables

**Table 1:** Demographic and clinical characteristics of study participants.

Characteristic	Control Group (n = 22)	PMS Group (n = 42)
Gender (male/female)	11/11 (50%/50%)	23/19 (54.8%/45.2%)
Median Age (years, range)	10 (6–14)	11 (7–15)
BMI (kg/m^2^, mean ± SD)	17.9 ± 2.2	18.2 ± 2.5
Gastrointestinal Symptoms (%)	2 (9.1%)	28 (66.7%)
Speech Delay (%)	0 (0%)	40 (95.2%)
Motor Coordination Issues (%)	0 (0%)	35 (83.3%)
Autistic Traits (%)	0 (0%)	38 (90.5%)

**Table 2:** Relative abundance of main bacterial phyla (bold) and genera in fecal samples from healthy controls and Phelan–McDermid syndrome (PMS) patients.

	Control Group		PMS Group		*p*-Value *
Phyla/Genera	N (%)	Median (IQR)	N (%)	Median (IQR)	
**Bacillota**	22 (100%)	88.28 (76.82–91.84)	40 (100%)	86.53 (80.96–91.70)	0.800
*Subdoligranulum*	22 (100%)	8.24 (6.42–15.05)	40 (100%)	5.57 (2.97–9.70)	0.024
*Faecalibacterium*	22 (100%)	9.82 (4.93–12.36)	40 (100%)	2.13 (1.16–7.35)	<0.001
*Blautia*	22 (100%)	5.41 (3.44–6.96)	40 (100%)	4.37 (3.28–7.03)	0.530
*Agathobacter*	22 (100%)	9.58 (1.80–12.61)	40 (100%)	1.33 (0.43–4.72)	0.011
*Dialister*	22 (100%)	3.69 (0.24–10.96)	32 (80%)	0.13 (<0.01–4.43)	0.031
*Ruminococcus*	22 (100%)	1.56 (0.34–4.40)	39 (97.5%)	1.46 (0.25–5.77)	0.910
*Ruminococcus*_*torques*_group	22 (100%)	0.64 (0.44–1.05)	40 (100%)	1.03 (0.33–2.28)	0.300
*Eubacterium hallii* group	22 (100%)	1.11 (0.73–2.29)	40 (100%)	3.00 (0.82–5.54)	0.014
*Eubacterium coprostanoligenes* group	21 (95.45%)	1.16 (0.57–3.17)	39 (97.5%)	1.83 (1.09–3.16)	0.210
*Anaerostipes*	22 (100%)	1.36 (0.53–2.24)	40 (100%)	2.06 (1.00–5.16)	0.110
*Dorea*	22 (100%)	1.78 (0.93–3.37)	38 (95%)	2.76 (1.11–4.15)	0.310
*Christensenellaceae R7* group	20 (90.91%)	0.96 (0.57–2.19)	38 (95%)	1.84 (0.61–3.59)	0.130
*Coprococcus*	22 (100%)	1.52 (1.02–2.78)	39 (97.5%)	1.59 (0.89–1.96)	0.620
*Roseburia*	22 (100%)	1.59 (0.95–3.24)	39 (97.5%)	0.83 (0.37–1.59)	0.007
*Streptococcus*	22 (100%)	0.58 (0.35–1.33)	40 (100%)	0.78 (0.19–1.76)	0.940
**Bacteroidota**	22 (100%)	6.91 (3.22–15.25)	40 (100%)	2.78 (1.05–6.79)	0.014
*Bacteroides*	22 (100%)	4.59 (2.57–7.33)	40 (100%)	1.51 (0.36–4.66)	0.004
*Alistipes*	22 (100%)	1.41 (0.45–3.79)	39 (97.5%)	0.55 (0.16–2.24)	0.038
**Actinomycetota**	22 (100%)	2.31 (1.45–4.27)	40 (100%)	3.92 (2.45–9.71)	0.030
*Bifidobacterium*	22 (100%)	1.22 (0.85–2.53)	40 (100%)	1.98 (0.98–6.38)	0.300
**Pseudomonadota**	22 (100%)	0.51 (0.32–0.84)	40 (100%)	0.36 (0.11–0.89)	0.150
**Verrucomicrobiota**	16 (72.73%)	0.07 (<0.01–0.28)	28 (70%)	0.13 (<0.01–1.12)	0.280
**Minor_phyla**	22 (100%)	0.07 (0.03–0.25)	40 (100%)	0.18 (0.09–0.41)	0.120
Minor_genera	22 (100%)	13.76 (11.37–17.79)	40 (100%)	20.52 (14.72–25.03)	0.002
Unclassified_genera	22 (100%)	11.74 (8.97–19.11)	40 (100%)	17.27 (12.10–25.35)	0.056

The prevalence is expressed as the number (percentage) of samples in which the bacterial taxa were detected and the relative abundance of the bacterial taxa as the median and the interquartile range (IQR). * Wilcoxon rank sum tests, with Bonferroni adjustment, to evaluate differences in the relative abundance of the phylum or genus.

**Table 3:** Concentration (μg/g) of fecal fatty acids (FAs) in the feces of the study participants.

Fatty Acids (μg/g, Mean ± SD)	Control Group	PMS Group	*p*-Value *
Acetic acid	3259.77 ± 84.85	2964.60 ± 105.16	>0.001
Propionic	1122.14 ± 90.53	861.67 ± 73.53	>0.001
Butyric	871.36 ± 54.10	668.52 ± 46.03	>0.001

* The *p*-values indicate the statistical significance of the differences between the two groups, as determined by Student’s *t*-tests.

**Table 4:** Frequencies of detection and concentrations of the immune factors in the fecal samples analyzed in this work. All the concentrations are expressed as ng/L.

	Control Group		PMS Group		*p*-Value *
	n (%)	Mean (sd)	n (%)	Mean (sd)	
IL-1β	8 (36.36%)	1.04 (2.01)	14 (33.33%)	2.71 (4.92)	0.44
IL-1ra	17 (77.27%)	831.60 (1495.05)	37 (88.10%)	993.68 (1589.26)	0.69
IL-4	2 (9.09%)	0.21 (0.37)	2 (4.76%)	0.29 (0.74)	0.62
IL-6	11 (50%)	0.88 (1.13)	23 (54.76%)	0.78 (1.03)	0.73
IL-8	1 (4.55%)	0.91 (0.001)	0 (0%)	0.95 (0.19)	0.32
IL-9	4 (18.18%)	1.86 (4.15)	1 (2.38%)	3.20 (5.08)	0.29
IL-10	16 (72.73%)	2.90 (5.07)	23 (54.76%)	4.09 (7.68)	0.52
IL-12 (p70)	20 (90.91%)	20.09 (13.67)	41 (97.62%)	20.10 (19.72)	0.99
IL-13	8 (36.36%)	1.21 (1.60)	13 (30.95%)	1.27 (1.19)	0.86
IL-17	17 (77.27%)	3.62 (6.18)	31 (73.81%)	6.04 (9.19)	0.28
Eotaxin	2 (9.09%)	0.18 (0.19)	4 (9.52%)	0.18 (0.15)	0.88
FGF basic	1 (4.55%)	18.36 (17.85)	3 (7.14%)	19.09 (19.55)	0.88
G-CSF	3 (13.64%)	14.79 (29.64)	3 (7.14%)	20.13 (44.23)	0.61
GM-CSF	1 (4.55%)	2.22 (2.19)	6 (14.29%)	1.95 (1.87)	0.61
IFN-γ	17 (77.27%)	11.70 (13.76)	27 (64.28%)	13.59 (11.56)	0.57
IP-10	5 (22.73%)	12.42 (7.58)	6 (14.29%)	13.98 (8.32)	0.47
MCP-1 (MCAF)	1 (4.55%)	3.889 (0.59)	1 (2.38%)	4.68 (4.18)	0.38
MIP-1a	7 (31.82%)	0.54 (0.49)	5 (11.90%)	0.81 (0.74)	0.13
PDGF-bb	21 (95.45%)	92.77 (75.49)	40 (95.24%)	97.45 (45.27)	0.76
MIP-1b	3 (13.64%)	1.17 (3.51)	4 (9.52%)	1.28 (3.55)	0.91
RANTES	21 (95.45%)	91.86 (21.42)	41 (97.62%)	95.14 (22.47)	0.58
TNF-α	7 (31.82%)	14.26 (57.12)	12 (28.57%)	28.97 (72.42)	0.41
VEGF	1 (4.55%)	835.78 (0.001)	0 (0%)	873.77 (178.19)	0.33

* The *p*-values indicate the statistical significance of the differences between the two groups, as determined by Student’s *t*-tests.

## Data Availability

The 16S rRNA gene sequences analyzed in this study are available in the NCBI repository under the accession code PRJNA1155863. Further inquiries can be directed to the corresponding authors.
